# 3,3′,5,5′-Tetra­nitro­biphen­yl

**DOI:** 10.1107/S1600536809011088

**Published:** 2009-04-18

**Authors:** N. Hammond, P. Carvalho, Y. Wu, M. A. Avery

**Affiliations:** a University of Mississippi, Department of Medicinal Chemistry, 417 Faser Hall, University, MS 38677, USA; b National Center for Natural Products Research, Research Institute of Pharmaceutical Sciences, School of Pharmacy, University of Mississippi, University, MS 38677, USA; c Department of Chemistry and Biochemistry, University of Mississippi, University, MS 38677, USA

## Abstract

The title compound, C_12_H_6_N_4_O_8_, is a biphenyl system that was synthesized as a building block for a new series of anti­malarial compounds. The aromatic rings are oriented at a dihedral angle of 45.5 (2)°, and inter­molecular short O⋯O contacts form a chain along the *b* axis. The strength of the inter­actions involved in this chain cause one of the rings to be slightly distorted, with the torsion angle between the nitro groups being 23.4 (2)°, whereas, in the other ring, both nitro systems are parallel, forming an angle of 9.6 (2)° with the plane of the aromatic ring to which they are bound. Furthermore, the three ring C atoms around the ring–ring linkage belong to a plane inclined by 4.5 (1)° in relation to the plane containing the other three C atoms, i.e. (NO_2_–)C—C—C(NO_2_). This distortion of the ring causes uncommonly short intermolecular O⋯O [3.038 (2) Å] and O⋯C [3.000 (4) and 3.214 (1) Å] contacts.

## Related literature

For the previous synthesis of the title compound and its stability studies, see Case (1942 ▶) and Hoffsommer & McCullough (1968 ▶). For the use of polynitro­aromatic compounds as explosives, see Davis (1941 ▶) and Keshavarz & Pouretedal (2005 ▶). For their mutagenic and carcinogenic properties, see Debnath *et al.* (1991 ▶). For previous studies showing distortions induced in aromatic rings, see Murray-Rust (1982 ▶), Allen *et al.* (1998 ▶), and Khrustalev *et al.* (2005 ▶). For the preparation, see: Goossen *et al.* (2007 ▶).
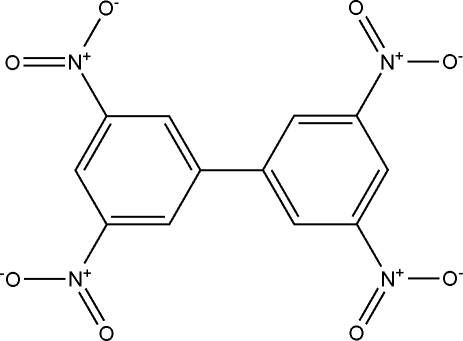


## Experimental

### 

#### Crystal data

C_12_H_6_N_4_O_8_
*M* *_r_* = 334.21
Orthorhombic, 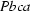

*a* = 10.0683 (1) Å
*b* = 15.4640 (2) Å
*c* = 16.3436 (2) Å
*V* = 2544.64 (5) Å^3^
*Z* = 8
Cu *K*α radiation
μ = 1.32 mm^−1^
*T* = 100 K
0.18 × 0.15 × 0.11 mm

#### Data collection

Bruker APEXII CCD diffractometer
Absorption correction: none
30954 measured reflections
2315 independent reflections
2294 reflections with *I* > 2σ(*I*)
*R*_int_ = 0.021

#### Refinement

*R*[*F* ^2^ > 2σ(*F* ^2^)] = 0.030
*wR*(*F* ^2^) = 0.081
*S* = 1.09
2315 reflections
225 parameters
H atoms treated by a mixture of independent and constrained refinement
Δρ_max_ = 0.22 e Å^−3^
Δρ_min_ = −0.31 e Å^−3^

### 

Data collection: *APEX2* (Bruker, 2008 ▶); cell refinement: *SAINT* (Bruker, 2005 ▶); data reduction: *SAINT*; program(s) used to solve structure: *SHELXS97* (Sheldrick, 2008 ▶); program(s) used to refine structure: *SHELXL97* (Sheldrick, 2008 ▶); molecular graphics: *SHELXTL* (Sheldrick, 2008 ▶); software used to prepare material for publication: *SHELXTL*.

## Supplementary Material

Crystal structure: contains datablocks I, global. DOI: 10.1107/S1600536809011088/im2105sup1.cif

Structure factors: contains datablocks I. DOI: 10.1107/S1600536809011088/im2105Isup2.hkl

Additional supplementary materials:  crystallographic information; 3D view; checkCIF report

## Figures and Tables

**Fig. 1.**
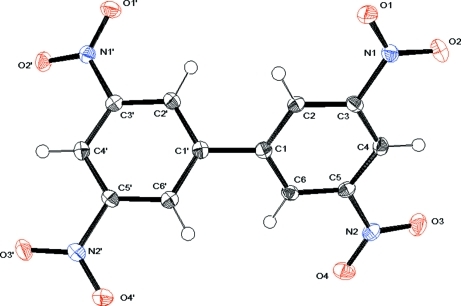
Molecular structure of the title compound, with the atom-numbering scheme. Displacement ellipsoids are drawn at the 50% probability level.

**Fig. 2.**
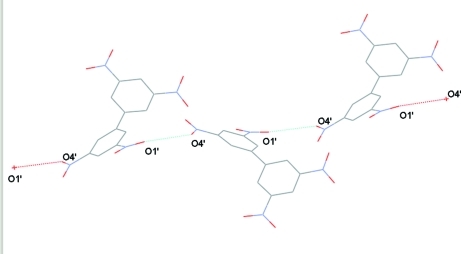
Partial view of the crystal packing of the title compound viewed along the b axis, showing molecules linked into chains by short intermolecular O1'···O4' interactions.

## supplementary crystallographic information

### Comment

Synthesis of the 3,3',5,5'-tetranitrobiphenyl has previously been reported by Case (1942), from the reaction of 3,5-dinitroiodobenzene and copper at 270°C. This paper reports the crystal structure of the title compound, obtained by reaction of 1-bromo-3,5-dinitrobenzene and 3,5-dinitrobenzoic acid in a sealed microwave tube in 1,2-dimethoxyethane. It was found to have activity against *Plasmodium falciparum *with an IC_50_ of 5.7 µ*M* against the chloroquine resistant D6 clone and 3.9µ*M* against the W2 clone.

### Experimental

The title compound was prepared by decarboxylative coupling as previously published (Goossen *et al.*, 2007). Briefly, 1-bromo-3,5-dinitrobenzene (247 mg, 1 mmol), 3,5-dinitrobenzoic acid (211 mg, 1 mmol), copper(II) bromide (180 mg, 0.8 mmol), 1,2-dimethoxyethane (3 ml), [tetrakis(triphenylphosphine)palladium(0)] (100 mg, 0.086 mmol), [dichlorobis(triphenylphospine)palladium (II)] (60 mg, 0.86 mmol), potassium carbonate (495 mg, 3 mmol) and water (1 ml) were added together in a microwave vial and microwaved at 160°C for 30 minutes while stirring, in a Biotage Initiator^TM^ Sixty, with variable microwave output. To keep the programmed temperature, the initial output was 100 W for 5 minutes, then varying between 0 and 30 W for the next 25 minutes. The reaction mixture was shaken 3 times with water, then brine and dried over magnesium sulfate and concentrated. The resulting residue was purified by column chromatography on silica gel, eluting with ethyl acetate/hexanes (90:10) to afford 62 mg (20% yield) of the title compound. 3,3',5,5'-Tetranitrobiphenyl was recrystallized from MeOH:CHCl_3_ (5:95) by slow evaporation of the solvent at room temperature.

### Refinement

All H atoms were located in difference maps, and treated as riding atoms, except H2' (C—H = 0.95 Å) and H4' (C—H = 0.94 Å), with the following distance restraints: C—H = 0.93 Å, *U*iso=1.2*U*eq (C) for C*sp*2.

### Crystal data

**Table:**

C_12_H_6_N_4_O_8_	*D*_x_ = 1.745 Mg m^−^^3^
*M**_r_* = 334.21	Melting point: not measured K
Orthorhombic, *P**b**c**a*	Cu *K*α radiation, λ = 1.54178 Å
Hall symbol: -P 2ac 2ab	Cell parameters from 9828 reflections
*a* = 10.0683 (1) Å	θ = 3.9–67.6°
*b* = 15.4640 (2) Å	µ = 1.32 mm^−^^1^
*c* = 16.3436 (2) Å	*T* = 100 K
*V* = 2544.64 (5) Å^3^	Block, yellow
*Z* = 8	0.18 × 0.15 × 0.11 mm
*F*(000) = 1360	

### Data collection

**Table:**

Bruker APEXII CCD diffractometer	2294 reflections with *I* > 2σ(*I*)
Radiation source: fine-focus sealed tube	*R*_int_ = 0.021
graphite	θ_max_ = 68.0°, θ_min_ = 5.4°
φ and ω scans	*h* = −12→12
30954 measured reflections	*k* = −18→18
2315 independent reflections	*l* = −19→19

### Refinement

**Table:**

Refinement on *F*^2^	Primary atom site location: structure-invariant direct methods
Least-squares matrix: full	Secondary atom site location: difference Fourier map
*R*[*F*^2^ > 2σ(*F*^2^)] = 0.030	Hydrogen site location: inferred from neighbouring sites
*wR*(*F*^2^) = 0.081	H atoms treated by a mixture of independent and constrained refinement
*S* = 1.09	*w* = 1/[σ^2^(*F*_o_^2^) + (0.0431*P*)^2^ + 1.2464*P*] where *P* = (*F*_o_^2^ + 2*F*_c_^2^)/3
2315 reflections	(Δ/σ)_max_ < 0.001
225 parameters	Δρ_max_ = 0.22 e Å^−^^3^
0 restraints	Δρ_min_ = −0.31 e Å^−^^3^
0 constraints	

### Special details

**Table:**

Geometry. All e.s.d.'s (except the e.s.d. in the dihedral angle between two l.s. planes) are estimated using the full covariance matrix. The cell e.s.d.'s are taken into account individually in the estimation of e.s.d.'s in distances, angles and torsion angles; correlations between e.s.d.'s in cell parameters are only used when they are defined by crystal symmetry. An approximate (isotropic) treatment of cell e.s.d.'s is used for estimating e.s.d.'s involving l.s. planes.
Refinement. Refinement of *F*^2^ against ALL reflections. The weighted *R*-factor *wR* and goodness of fit *S* are based on *F*^2^, conventional *R*-factors *R* are based on *F*, with *F* set to zero for negative *F*^2^. The threshold expression of *F*^2^ > σ(*F*^2^) is used only for calculating *R*-factors(gt) *etc*. and is not relevant to the choice of reflections for refinement. *R*-factors based on *F*^2^ are statistically about twice as large as those based on *F*, and *R*- factors based on ALL data will be even larger.

### Fractional atomic coordinates and isotropic or equivalent isotropic displacement parameters (Å^2^)

**Table:**

	*x*	*y*	*z*	*U*_iso_*/*U*_eq_	
C1	0.34665 (12)	0.30780 (8)	0.43269 (7)	0.0157 (3)	
C2	0.33233 (12)	0.30138 (8)	0.51737 (7)	0.0156 (3)	
H2	0.2894	0.3448	0.5466	0.019*	
C3	0.38272 (12)	0.22966 (8)	0.55741 (7)	0.0162 (3)	
C4	0.44841 (12)	0.16333 (8)	0.51778 (8)	0.0168 (3)	
H4	0.4828	0.1160	0.5458	0.020*	
C5	0.45990 (12)	0.17162 (8)	0.43369 (8)	0.0165 (3)	
C6	0.41093 (12)	0.24155 (8)	0.39006 (7)	0.0162 (3)	
H6	0.4206	0.2443	0.3335	0.019*	
N1	0.37026 (10)	0.22503 (7)	0.64699 (6)	0.0182 (2)	
N2	0.52978 (10)	0.10245 (7)	0.38839 (7)	0.0189 (2)	
O1	0.30268 (11)	0.28031 (6)	0.68111 (5)	0.0259 (2)	
O2	0.42893 (10)	0.16675 (6)	0.68273 (5)	0.0236 (2)	
O3	0.58781 (10)	0.04674 (6)	0.42849 (6)	0.0276 (2)	
O4	0.52572 (10)	0.10442 (6)	0.31365 (6)	0.0253 (2)	
C1'	0.30389 (12)	0.38719 (8)	0.38844 (7)	0.0155 (3)	
C2'	0.33837 (12)	0.46789 (8)	0.42007 (7)	0.0162 (3)	
H2'	0.3828 (15)	0.4741 (10)	0.4712 (9)	0.018 (4)*	
C3'	0.31428 (12)	0.54092 (8)	0.37355 (7)	0.0156 (3)	
C4'	0.25353 (12)	0.53897 (8)	0.29759 (7)	0.0159 (3)	
H4'	0.2391 (15)	0.5895 (9)	0.2666 (9)	0.018 (4)*	
C5'	0.21448 (12)	0.45823 (8)	0.27031 (7)	0.0160 (3)	
C6'	0.23899 (12)	0.38234 (8)	0.31302 (7)	0.0161 (3)	
H6'	0.2127	0.3292	0.2918	0.019*	
N1'	0.36202 (10)	0.62466 (7)	0.40437 (6)	0.0169 (2)	
N2'	0.14792 (10)	0.45304 (7)	0.18984 (6)	0.0177 (2)	
O1'	0.38516 (9)	0.63076 (6)	0.47790 (5)	0.0231 (2)	
O2'	0.37639 (9)	0.68355 (6)	0.35486 (6)	0.0212 (2)	
O3'	0.12418 (9)	0.52096 (6)	0.15399 (5)	0.0233 (2)	
O4'	0.12047 (10)	0.38108 (6)	0.16341 (6)	0.0246 (2)	

### Atomic displacement parameters (Å^2^)

**Table:**

	*U*^11^	*U*^22^	*U*^33^	*U*^12^	*U*^13^	*U*^23^
C1	0.0138 (6)	0.0161 (6)	0.0173 (6)	−0.0029 (5)	−0.0013 (4)	0.0007 (5)
C2	0.0155 (6)	0.0143 (6)	0.0171 (6)	−0.0015 (5)	0.0002 (5)	−0.0013 (5)
C3	0.0170 (6)	0.0179 (6)	0.0137 (6)	−0.0044 (5)	−0.0009 (4)	0.0009 (5)
C4	0.0162 (6)	0.0149 (6)	0.0193 (6)	−0.0018 (5)	−0.0024 (5)	0.0028 (5)
C5	0.0148 (6)	0.0154 (6)	0.0192 (6)	−0.0021 (5)	0.0010 (5)	−0.0025 (5)
C6	0.0163 (6)	0.0183 (6)	0.0140 (6)	−0.0035 (5)	−0.0005 (4)	−0.0003 (5)
N1	0.0214 (5)	0.0174 (5)	0.0158 (5)	−0.0039 (4)	0.0003 (4)	0.0015 (4)
N2	0.0167 (5)	0.0169 (5)	0.0230 (6)	−0.0012 (4)	0.0017 (4)	−0.0019 (4)
O1	0.0409 (6)	0.0193 (5)	0.0175 (5)	0.0015 (4)	0.0061 (4)	−0.0017 (4)
O2	0.0248 (5)	0.0269 (5)	0.0191 (5)	0.0004 (4)	−0.0015 (4)	0.0084 (4)
O3	0.0309 (5)	0.0200 (5)	0.0320 (5)	0.0079 (4)	0.0017 (4)	0.0022 (4)
O4	0.0263 (5)	0.0304 (5)	0.0193 (5)	0.0025 (4)	0.0033 (4)	−0.0057 (4)
C1'	0.0137 (6)	0.0173 (6)	0.0155 (6)	0.0000 (4)	0.0026 (4)	0.0011 (5)
C2'	0.0146 (6)	0.0194 (6)	0.0147 (6)	0.0005 (5)	0.0003 (5)	0.0008 (5)
C3'	0.0138 (6)	0.0154 (6)	0.0177 (6)	−0.0008 (4)	0.0017 (5)	−0.0011 (5)
C4'	0.0138 (5)	0.0173 (6)	0.0166 (6)	0.0018 (5)	0.0024 (5)	0.0030 (5)
C5'	0.0133 (6)	0.0206 (6)	0.0141 (6)	0.0006 (5)	0.0001 (4)	0.0007 (5)
C6'	0.0154 (6)	0.0164 (6)	0.0165 (6)	−0.0005 (5)	0.0021 (5)	−0.0004 (5)
N1'	0.0144 (5)	0.0172 (5)	0.0192 (5)	0.0008 (4)	−0.0004 (4)	0.0003 (4)
N2'	0.0165 (5)	0.0211 (5)	0.0155 (5)	0.0003 (4)	−0.0004 (4)	0.0013 (4)
O1'	0.0283 (5)	0.0236 (5)	0.0175 (5)	−0.0042 (4)	−0.0031 (4)	−0.0022 (4)
O2'	0.0246 (5)	0.0154 (4)	0.0236 (5)	−0.0015 (4)	−0.0009 (4)	0.0042 (4)
O3'	0.0274 (5)	0.0222 (5)	0.0205 (5)	0.0013 (4)	−0.0059 (4)	0.0063 (4)
O4'	0.0314 (5)	0.0213 (5)	0.0210 (5)	−0.0028 (4)	−0.0074 (4)	−0.0024 (4)

### Geometric parameters (Å, °)

**Table:**

C1—C1'	1.4885 (17)	C5'—N2'	1.4783 (16)
C1'—C2'	1.3947 (17)	C5—N2	1.4790 (16)
C1'—C6'	1.3971 (18)	C6—C1	1.3978 (17)
C2—C1	1.3950 (17)	C6—C5	1.3860 (17)
C2—C3	1.3842 (17)	C6—H6	0.9300
C2—H2	0.9300	C6'—H6'	0.9300
C2'—H2'	0.953 (15)	N1'—C3'	1.4702 (15)
C3'—C2'	1.3828 (17)	N1—O1	1.2266 (14)
C3—C4	1.3816 (18)	N1'—O1'	1.2278 (14)
C3—N1	1.4712 (15)	N1—O2	1.2257 (14)
C4'—C3'	1.3843 (17)	N1'—O2'	1.2268 (14)
C4—H4	0.9300	N2'—O3'	1.2263 (14)
C4'—H4'	0.942 (15)	N2—O3	1.2301 (14)
C5'—C4'	1.3828 (17)	N2—O4	1.2226 (15)
C5—C4	1.3851 (18)	N2'—O4'	1.2252 (14)
C5'—C6'	1.3876 (17)		
			
C1—C2—H2	120.4	C4—C5—N2	118.00 (11)
C1'—C2'—H2'	122.1 (9)	C5'—C4'—C3'	115.76 (11)
C1'—C6'—H6'	120.6	C5—C4—H4	122.1
C1—C6—H6	120.7	C5'—C4'—H4'	122.1 (9)
C2'—C1'—C1	119.08 (11)	C5—C6—C1	118.69 (11)
C2—C1—C1'	120.71 (11)	C5'—C6'—C1'	118.79 (11)
C2—C1—C6	119.36 (11)	C5'—C6'—H6'	120.6
C2'—C1'—C6'	119.44 (11)	C5—C6—H6	120.7
C2'—C3'—C4'	123.55 (11)	C6—C1—C1'	119.75 (11)
C2'—C3'—N1'	118.27 (11)	C6'—C1'—C1	121.31 (11)
C2—C3—N1	118.57 (11)	C6—C5—N2	118.42 (11)
C3'—C2'—C1'	118.90 (11)	C6'—C5'—N2'	118.84 (11)
C3—C2—C1	119.21 (11)	O1'—N1'—C3'	117.71 (10)
C3'—C2'—H2'	118.9 (9)	O1—N1—C3	117.76 (10)
C3—C2—H2	120.4	O2'—N1'—C3'	117.81 (10)
C3—C4—C5	115.88 (11)	O2—N1—C3	117.96 (10)
C3—C4—H4	122.1	O2—N1—O1	124.27 (10)
C3'—C4'—H4'	122.2 (9)	O2'—N1'—O1'	124.48 (11)
C4—C3—C2	123.28 (11)	O3—N2—C5	117.76 (10)
C4'—C3'—N1'	118.09 (10)	O3'—N2'—C5'	117.84 (10)
C4—C3—N1	118.11 (11)	O4'—N2'—C5'	117.72 (10)
C4'—C5'—C6'	123.42 (11)	O4—N2—C5	117.79 (10)
C4—C5—C6	123.57 (11)	O4'—N2'—O3'	124.44 (10)
C4'—C5'—N2'	117.70 (10)	O4—N2—O3	124.45 (11)
			
C1—C1'—C2'—C3'	171.43 (11)	C5'—C4'—C3'—C2'	1.28 (18)
C1—C1'—C6'—C5'	−172.98 (11)	C5'—C4'—C3'—N1'	177.92 (10)
C1—C2—C3—C4	0.57 (18)	C5—C6—C1—C1'	174.55 (11)
C1—C2—C3—N1	178.25 (11)	C5—C6—C1—C2	−0.67 (17)
C1—C6—C5—C4	0.20 (18)	C6—C1—C1'—C2'	−129.65 (12)
C1—C6—C5—N2	−178.99 (10)	C6—C1—C1'—C6'	45.44 (17)
C2—C1—C1'—C2'	45.51 (17)	C6'—C1'—C2'—C3'	−3.76 (18)
C2—C1—C1'—C6'	−139.40 (12)	C6—C5—C4—C3	0.62 (18)
C2'—C1'—C6'—C5'	2.09 (18)	C6'—C5'—C4'—C3'	−3.07 (18)
C2—C3—C4—C5	−1.01 (18)	C6—C5—N2—O3	170.62 (11)
C2—C3—N1—O1	7.99 (17)	C6'—C5'—N2'—O3'	178.23 (11)
C2—C3—N1—O2	−171.36 (11)	C6'—C5'—N2'—O4'	−1.94 (17)
C3—C2—C1—C1'	−174.87 (11)	C6—C5—N2—O4	−9.53 (16)
C3—C2—C1—C6	0.31 (18)	N1'—C3'—C2'—C1'	−174.56 (11)
C4'—C3'—C2'—C1'	2.08 (19)	N1—C3—C4—C5	−178.70 (10)
C4—C3—N1—O1	−174.20 (11)	N2'—C5'—C4'—C3'	179.39 (10)
C4—C3—N1—O2	6.44 (16)	N2—C5—C4—C3	179.81 (10)
C4'—C5'—C6'—C1'	1.44 (19)	N2'—C5'—C6'—C1'	178.95 (10)
C4'—C5'—N2'—O3'	−4.12 (16)	O1'—N1'—C3'—C2'	−20.79 (16)
C4—C5—N2—O3	−8.62 (16)	O1'—N1'—C3'—C4'	162.39 (11)
C4—C5—N2—O4	171.23 (11)	O2'—N1'—C3'—C2'	158.99 (11)
C4'—C5'—N2'—O4'	175.71 (11)	O2'—N1'—C3'—C4'	−17.83 (16)
